# Impact of autoantibodies against the M2-muscarinic acetylcholine receptor on clinical outcomes in peripartum cardiomyopathy patients with standard treatment

**DOI:** 10.1186/s12872-021-02414-7

**Published:** 2021-12-28

**Authors:** Guiling Ma, Long Chen, Yin Yue, Xiyan Liu, Yidan Wang, Cheng Shi, Fei Song, Wei Shi, Yingshih Lo, Lin Zhang

**Affiliations:** 1 Department of Cardiology, Beijing Key Laboratory of Hypertension Research, Beijing Chao-Yang Hospital, Capital Medical University, 8# Gong-Ti South Road, Chaoyang District, Beijing, 100020 China; 2 HTRM Cardiologist Group, BENQ Medical Center, Nanjing Medical University, 181# Zhuyuan Road, Suzhou City, 215000 JiangSu Province China

**Keywords:** Anti-M2-muscarinic receptor, Peripartum cardiomyopathy, Vagus nerve system

## Abstract

**Objectives:**

To evaluate the impact of autoantibodies against the M2-muscarinic receptor (anti-M2-R) on the clinical outcomes of patients receiving the standard treatment for peripartum cardiomyopathy (PPCM).

**Methods:**

A total of 107 PPCM patients who received standard heart failure (HF) treatment between January 1998 and June 2020 were enrolled in this study. According to anti-M2-R reactivity, they were classified into negative (n = 59) and positive (n = 48) groups, denoted as the anti-M2-R (−) and anti-M2-R (+) groups. Echocardiography, 6-min walk distance, serum digoxin concentration (SDC), and routine laboratory tests were performed regularly for 2 years. The all-cause mortality, cardiovascular mortality, and rehospitalisation rate for HF were compared between the two groups.

**Results:**

A total of 103 patients were included in the final data analysis, with 46 in the anti-M2-R (+) group and 57 in the anti-M2-R (−) group. Heart rate was lower in the anti-M2-R (+) group than in the anti-M2-R (−) group at the baseline (102.7 ± 6.1 bpm vs. 96.0 ± 6.4 bpm, *p* < 0.001). The initial SDC was higher in the anti-M2-R (+) group than in the anti-M2-R (−) group with the same dosage of digoxin (1.25 ± 0.45 vs. 0.78 ± 0.24 ng/mL, *p* < 0.001). The dosages of metoprolol and digoxin were higher in the anti-M2-R (−) patients than in the anti-M2-R (+) patients (38.8 ± 4.6 mg b.i.d. vs. 27.8 ± 5.3 mg b.i.d., *p* < 0.0001, respectively, for metoprolol; 0.12 ± 0.02 mg/day vs. 0.08 ± 0.04 mg/day, *p* < 0.0001, respectively, for digoxin). Furthermore, there was a greater improvement in cardiac function in the anti-M2-R (−) patients than in the anti-M2-R (+) patients. Multivariate analysis identified negativity for anti-M2-R as the independent predictor for the improvement of cardiac function. Rehospitalisation for HF was lower in the anti-M2-R (−) group, but all-cause mortality and cardiovascular mortality were the same.

**Conclusions:**

There were no differences in all-cause mortality or cardiovascular mortality between the two groups. Rehospitalisation rate for HF decreased in the anti-M2-R (−) group. This difference may be related to the regulation of the autonomic nervous system by anti-M2-R.

## Introduction

Peripartum cardiomyopathy (PPCM) is a rare idiopathic dilated cardiomyopathy defined by the signs and symptoms of heart failure (HF) in the last month of pregnancy through the fifth month postpartum [1]. The definition of PPCM requires that there is no previously known structural heart disease, and that echocardiographic parameters achieve left ventricular ejection fraction (LVEF) < 45% and/or fractional shortening < 30%, with a possible additive left ventricular end-diastolic dimension > 2.7 cm/m^2^ body surface area [2]. This disease is associated with high morbidity and mortality, yet its aetiology remains unknown [1].

Autoimmune mechanisms have been shown to play a role in the pathogenesis of PPCM. Autoantibodies against β1 adrenergic receptors (anti-β1-R) and autoantibodies against M2-muscarinic receptor (anti-M2-R) are more common in patients with PPCM [3]. These autoantibodies could can interfere with radioligand binding on the target receptor, reveal various agonist-like activities on the corresponding cardiac receptors, and, hence, modulate cardiac function [4]. Our previous study found the positive rate was 59.5% for anti-β1-R and 45.9–47.5% for anti-M2-R in PPCM patients [3, 5]. However, the clinical significance of these findings is currently unknown.

The roles of excessive activation of the renin–angiotensin–aldosterone system (RAAS) and the sympathetic nervous system have been recognised in the pathogenesis of HF by most doctors. However, the importance of the vagus nerve system is relatively unfamiliar. The M2-muscarinic acetylcholine receptor is the most important cardiac receptor of the vagus nerve system. What is the clinical significance of anti-M2-R? In this study, the connection between the presence of anti-M2-R and cardiac function was investigated in PPCM patients who received the standard treatment regimen for HF [angiotensin converting enzyme inhibitor (ACEI), β-receptor blocker, furosemide, and spironolactone]. The primary endpoints were all-cause mortality, cardiovascular mortality, and rehospitalisation for HF.

## Methods

### Study population

This was a prospective observational study, which began in January 1998 and ended in December 2020. A total of 107 consecutively newly diagnosed PPCM patients who received the standard treatment regimen for HF were enrolled at Beijing Chao-Yang Hospital and BENQ Medical Centre. Demographic data and related information were obtained by in-person interviews using a structured questionnaire. The inclusion criteria were as follows: (1) age between 18 and 40 years old; (2) heart function of New York Heart Association functional classes (NYHA) II–IV; (3) symptoms of HF occurring in the last month of pregnancy or during the first 5 months postpartum; and (4) no other identifiable causes of HF. The exclusion criteria were as follows: (1) clinical conditions with increased levels of autoantibodies, such as rheumatoid arthritis, HIV, and sepsis; (2) moderate to severe anaemia; (3) metabolic disorders, such as thyroid disease; or (4) moderate to severe hepatic or renal dysfunction. The study protocol complied with the Declaration of Helsinki and was approved by the Ethics Committee of Beijing Chao-Yang Hospital and BENQ Medical Centre. All patients provided written informed consent before the study.

### Serum anti-M2-R detection

About 2 mL of blood was taken from the antecubital vein of each patient upon enrolment in the study. The serum samples were separated by centrifugation at 3000 rpm for 10 min and stored at − 20 °C. A peptide corresponding to the sequence of the second extracellular loop of human M2-muscarinic receptor (amino acid sequence number 169–193: V-R-T-V-E-D-G-E-C-Y-I-Q-F-F-S-N-A-A-V-T-F-G-T-A-I) was synthesised by Genomed (Genomed Synthesis, Inc., San Francisco, CA, USA) with the solid-phase method of Merrifield. The purity of the peptide was 98% based on high-performance liquid chromatography analysis on a Vydac C-18 column. The serum levels of anti-M2-R were measured with ELISA. Positive was defined as the ratio of (sample *A* − blank *A*)/(negative control *A* − blank *A*) ≥ 2.1 [6]. Titres of autoantibodies were the highest when this ratio was ≥2.1 with serum diluted from 1:20 to 1:160. The intra-assay and inter-assay coefficients of variation were less than 5%.

### Standard pharmacological regimen

The standard pharmacological regimen for HF was recommended for all the patients (perindopril or losartan, metoprolol, furosemide, and spironolactone). Patients diagnosed prenatally were given furosemide and metoprolol. Perindopril and spironolactone were added after delivery of the foetus. For patients with NYHA functional class III–IV, digoxin was prescribed at the beginning of the treatment; for patients with NYHA functional class II, digoxin was started when the symptoms persisted after 1 month of treatment with perindopril, metoprolol, furosemide, and spironolactone. The maximum tolerated heart rate and blood pressure were 60–75 bpm and 120/65 ± 10/5 mm Hg, respectively. Perindopril was taken at an initial dose of 1–2 mg/day and then uptitrated depending on the blood pressure. If perindopril was not tolerated, losartan was used instead. Metoprolol was taken at an initial dose of 12.5 mg/day, which was uptitrated over a 2–4-week period by doubling the twice-daily amount to the maximum tolerated dose or a target of 100 mg/day [2]. The initial dosage of furosemide was 10–20 mg/day, and it was increased if a patient showed signs or symptoms of HF progression. The dosage of spironolactone was 10–20 mg/day. Digoxin was taken at an initial dose of 0.125 mg/day and then adjusted according to the serum digoxin concentration (SDC). The target SDC was 0.5–0.9 ng/mL, as suggested by the ACCF/AHA Guideline for the Management of HF [7]. If the SDC was between 0.9 and 1.8 ng/mL, the dosage of digoxin was reduced to 0.0625 mg/day. The dosage of digoxin was reduced to 0.0625 mg every other day when the SDC was higher than 1.8 ng/mL. In addition, patients were advised to control their salt intake and body weight.

If the structure and function of the left ventricle returned to normal (LVEDD < 50 mm and LVEF > 50%) within 1 year, the administration of digoxin was stopped. If normalisation occurred during the second year, the administration of metoprolol, spironolactone, furosemide, and digoxin gradually was stopped, and only the administration of perindopril/losartan was continued.

### Follow-up examination

All patients were assigned to a fixed investigator for 2 years of follow-up. The primary endpoint events were all-cause mortality, cardiovascular mortality, and rehospitalisation for HF. Patients received a follow-up once a month for the first year and every 3–6 months for the second year. Echocardiography and 6-min walk tests were performed every 6 months. Clinical laboratory tests, including SDC, were conducted on the first month and every 3 months thereafter. Serum anti-M2-R testing was performed once a year. The data of heart rate, blood pressure, body weight, cardiac function, presence of peripheral oedema, and drug dosages were also recorded during the examinations. The subjects were questioned and examined for the presence of any adverse drug reactions.

### Statistical methods

Quantitative data are presented as the mean ± standard deviation (SD), and categorical data are presented as percentages. The titre of serum anti-M2-R is reported as the geometric mean. For comparison between the two groups, the Student’s *t-*test was used for continuous variables, and the chi-square test for categorical variables. Univariate and multivariate logistic regression analyses were performed to evaluate the association between anti-M2-R and rehospitalisation for HF, using the stepwise logistic regression model. Baseline variables that were considered clinically relevant or that showed a univariate relationship with outcome (*p* < 0.05) were included in the mulivariate analysis. Cut-offs for entry and departure in the logistic regression model were 0.05 and 0.10, respectively. In the above analysis, the odds ratio (OR) and 95% confidence intervals (CIs) were used to assess the factors related to risk of rehospitalisation for HF. Data on the titration of metoprolol were fit to a variable slope sigmoidal equation: Y = Initial Dose + (Maximum Dose − Initial Dose)/(1 + 10(LogEC50 − X)* Slope), where the independent variable (X) is the log of the time of the dosage value (Y), and LogEC50 denotes the time that corresponds to halfway between the minimum and maximum dosages. Chi-square statistics and log-rank test were used for all-cause mortality, cardiovascular mortality, and rehospitalisation for HF. All the tests were two-tailed. *p* < 0.05 was considered statistically significant. Data were analysed using SPSS 25.0 (SPSS, Chicago, Illinois, USA).

## Results

### Study characteristics

All 107 patients received the diagnosis of PPCM for the first time. Among them, 46 patients were primiparous, and 29 patients had multiple gestations. There were 41 patients with pregnancy-induced hypertension and 28 patients with gestational diabetes mellitus. There were 62 patients with symptoms in the postpartum period. At baseline, 35 patients were in NYHA functional class II, 50 patients were in class III, and 22 patients were in class IV.

According to the anti-M2-R reactivity, 59 patients were assigned to the negative group, denoted as the anti-M2-R (−) group, and the other 48 patients were assigned to the positive group, denoted as the anti-M2-R (+) group. The baseline characteristics of the two groups are shown in Table 1. Four patients were lost to follow-up during the first year (two patients in the anti-M2-R (−) group and two patients in the anti-M2-R (+) group), and the remaining 103 patients completed the final data analysis. There were 25 cases of HF with midrange ejection fraction (HFmrEF) in the anti-M2-R (+) group and 24 cases in the anti-M2-R (−) group. Of all the parameters, only the mean resting heart rate of the anti-M2-R (−) group was higher than that of the anti-M2-R (+) group. It was hypothesised that anti-M2-R may influence the heart rate via the activation of the vagus nerve system.

**Table 1:** Clinical characteristics of PPCM patients

	Anti-M2-R (+) group n = 48	Anti-M2-R (−) group n = 59	*p* value
Age (years)	30.1± 3.6	29.5 ± 3.7	0.383
Multiple gestation	14	15	0.665
Multiparity	28	33	0.803
Pregnancy complications			
Pregnancy-induced hypertension	19	22	0.808
Gestational diabetes mellitus	12	16	0.804
Postpartum diagnosed PPCM	29	33	0.640
Blood pressure (mm Hg)			
Systolic	137.2 ± 13.3	136.5 ± 14.5	0.779
Diastolic	88.0 ± 10.2	87.5 ± 10.7	0.797
Heart rate (bpm)	96.0 ± 6.4	102.7 ± 6.1	< 0.001
NYHA functional class	2.85 ± 0.73	2.88 ± 0.71	0.834
Echocardiographic data			
LVEDD (mm)	60.7 ± 5.6	60.4 ± 5.8	0.823
LVESD (mm)	47.3 ± 6.1	47.0 ± 6.7	0.809
LVEF (%)	38.0 ± 4.9	38.2 ± 3.2	0.763
HFmrEF	25	24	0.216
Six-minute walk distance (m)	195.9 ± 83.2	199.8 ± 78.0	0.802
NT-proBNP (pg/mL)	4017.2 ± 1583.3	4411.0 ± 1838.2	0.253
Creatine (µmol/L)	64.6 ± 10.6	66.7 ± 12.0	0.364

*PPCM* peripartum cardiomyopathy, *LVEDD* left ventricular end-diastolic diameter, *LVESD* left ventricular end-systolic diameter, *LVEF* left ventricular ejection fraction, *HFmrEF* HF with midrange ejection fraction, *NT-proBNP* N-terminal pro-brain natriuretic peptide

### Drug dosages

All patients, including the HFmrEF patients, received the standard therapy regimen for HF. In both groups, four patients were given losartan, and all the dosages were 50 mg/day. All the patients used spironolactone and furosemide. However, perindopril and/or metoprolol were discontinued in nine patients with hypotension and dizziness at the minimum dosage during the first year of follow-up. There were no differences between the two groups regarding the dosages of perindopril, spironolactone, or furosemide. The dosages of metoprolol and digoxin in the anti-M2-R (−) group were higher than those in the anti-M2-R (+) group, as shown in Table 2.

**Table 2:** Dosages of drugs

	Anti-M2-R (+) group	Anti-M2-R (−) group	*p* value
Perindopril, mg	2.9 ± 1.1	2.9 ± 1.0	0.776
Metoprolol, mg	27.8 ± 5.3	38.8 ± 4.6	< 0.001
Spironolactone, mg	18.5 ± 3.6	19.1 ± 2.9	0.328
Furosemide, mg	21.1 ± 6.4	20.7 ± 6.8	0.769
Digoxin, mg	0.08 ± 0.04	0.12 ± 0.02	< 0.001

*Anti-M2-R* autoantibodies against the M2-muscarinic receptor

### Titration of metoprolol

**Fig. 1:**
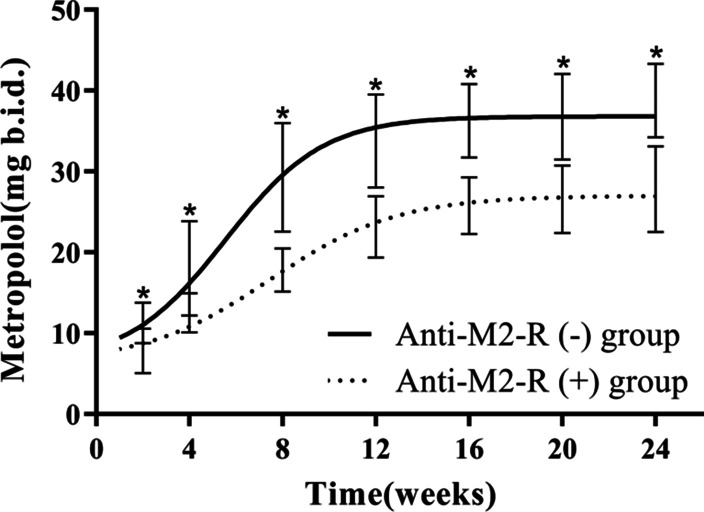
Non-linear fit of metoprolol titration data. *p < 0.001 for the comparison between the two groups

Patients in the anti-M2-R (−) group demonstrated a more rapid rate of uptitration and a higher dosage of metoprolol than those in the anti-M2-R (+) group. The maximum tolerated dosage of metoprolol for the anti-M2-R (−) group was 38.8 ± 4.6 mg b.i.d., which was higher than 27.8 ± 5.3 mg b.i.d. for the anti-M2-R (+) group (*p* < 0.001), as shown in Fig. 1. The mean time to maximum tolerated dosage of metoprolol was 67.3 ± 10.5 days in the anti-M2-R (−) group, which was shorter than 80.9 ± 10.6 days in the anti-M2-R (+) group (*p* < 0.001).

### Serum digoxin concentration and the dose of digoxin

Digoxin was prescribed at an initial dose of 0.125 mg/day. The mean SDC was significantly higher in anti-M2-R (+) patients than in anti-M2-R (−) patients for the first month of detection (1.25 ± 0.45 vs. 0.78 ± 0.24 ng/mL, *p* < 0.001). The dosage of digoxin was reduced depending on the SDC. The mean maintenance dose of digoxin was 0.12 ± 0.02 mg/day in the anti-M2-R (–) group, significantly higher than 0.08 ± 0.04 mg/day of the anti-M2-R (+) group (*p* < 0.001). Finally, there was no significant difference in the SDC levels between the two groups.

### Dynamic variation in serum anti-M2-R

**Fig. 2:**
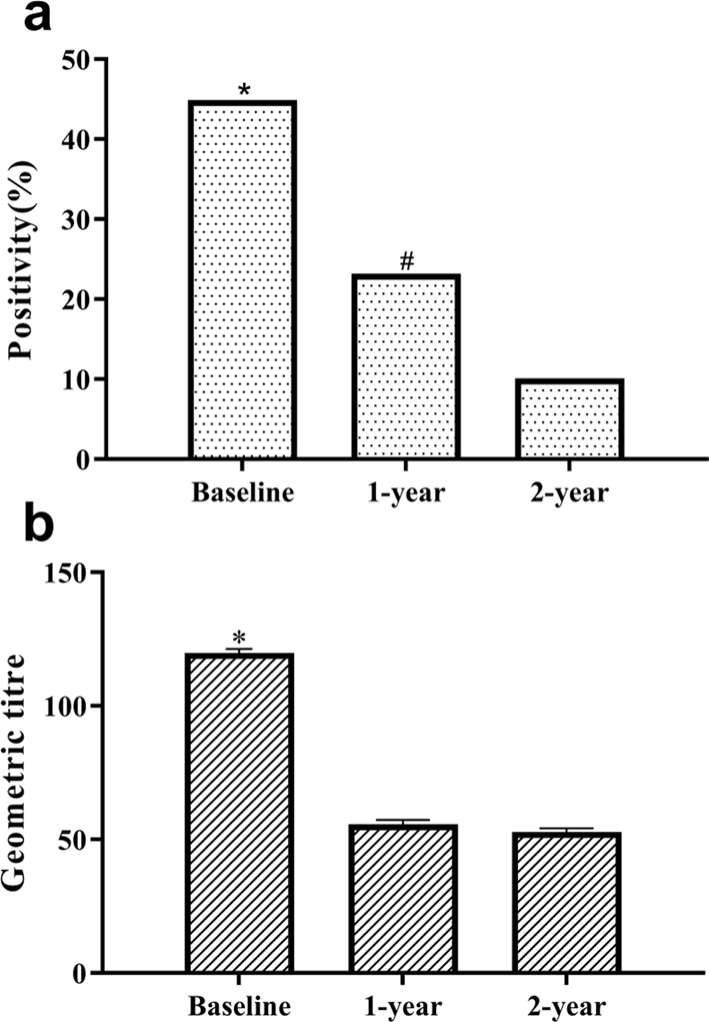
Frequencies and titres of serum anti-M2-R. ** a** Frequency. **b** Geometric titre. *p < 0.001 for the comparison of frequencies and geometric mean titres of anti-M2-R between the baseline, 1-year mark, and 2-year mark. ^#^*p* < 0.05 between 1- and 2-year marks

The serum was positive for anti-M2-R in 44.9% (48/107) of the PPCM patients at enrolment. In positive cases, the mean titre of anti-M2-R was 1:120. At the end of the first and second years after treatment, the positive rate of serum anti-M2-R dropped to 23.2% (23/99) and 10.1% (10/99), and the mean titre dropped to 1:56 and 1:53, respectively, compared with baseline (all *p* < 0.001) (Fig. 2).

### Adverse events

There were no obvious influences on blood glucose, serum lipid, hepatic function, or potassium level during the treatment or follow-up periods. Renal function was regularly monitored, and there was no significant fluctuation in the serum creatinine levels. Only one patient in the anti-M2-R (+) group showed symptoms of digoxin intoxication, such as slight weakness and nausea. The SDC of this patient was 2.5 ng/mL, so the dosage of digoxin was reduced to 0.0625 mg every other day, and the symptoms subsequently disappeared.

### Cardiac function and 6-min walk test

**Fig. 3:**
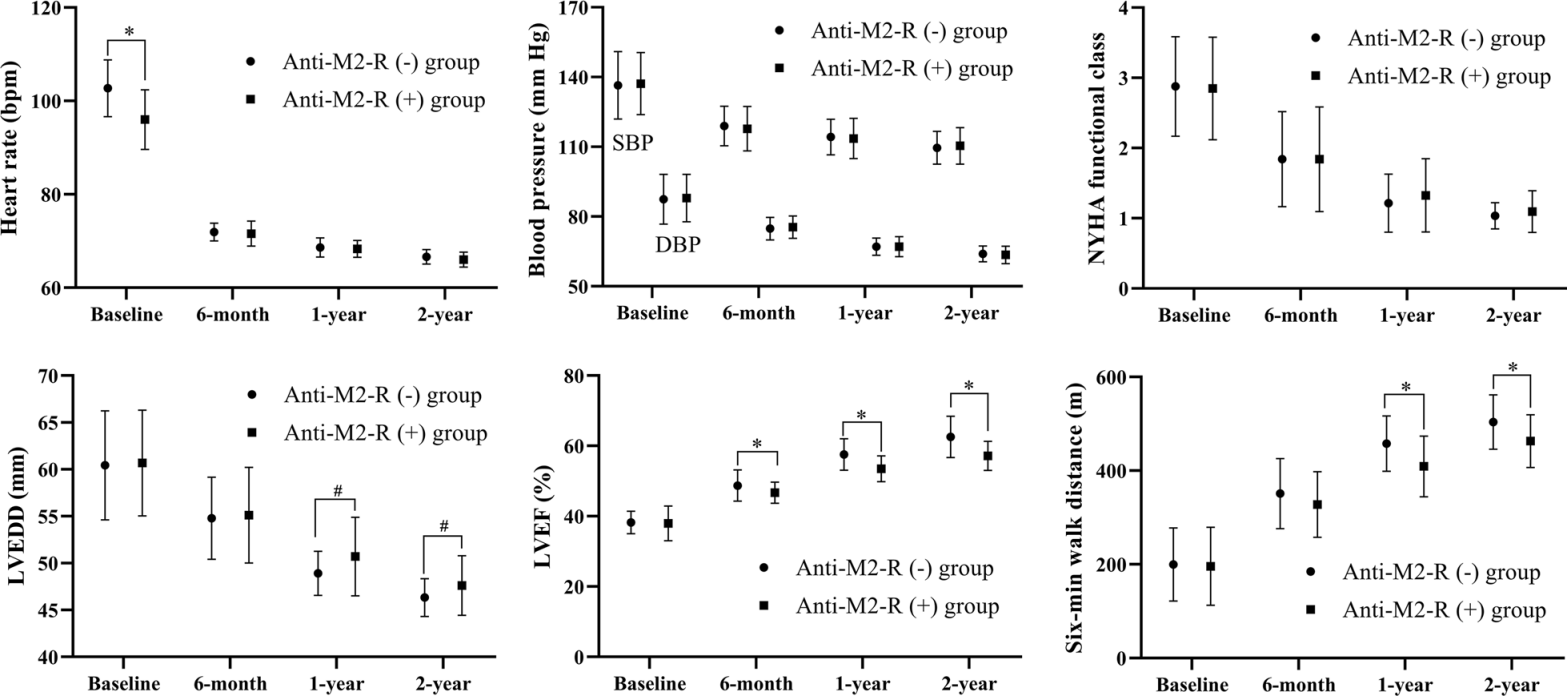
Comparison of the clinical indices. *p < 0.001 for the comparison between the two groups. ^#^*p* < 0.05 for the comparison between the two groups. *NYHA* New York Heart Association, *LVEDD* left ventricular end-diastolic diameter, *LVEF* left ventricular ejection fraction

The clinical data, NYHA functional class, echocardiographic results, and 6-min walk distance at the baseline, 6-month, 1-year, and 2-year marks were determined (Fig. 3). LVEDD decreased from 60.4 ± 5.8 to 46.3 ± 2.0 mm in the anti-M2-R (−) group and from 60.7 ± 5.6 to 47.6 ± 3.2 mm in the anti-M2-R (+) group. LVEF increased from 38.2 ± 3.2 to 62.6 ± 5.9% in the anti-M2-R (−) group and from 38.0 ± 4.9 to 57.2 ± 4.1% in the anti-M2-R (+) group. The 6-min walk distance increased from 199.8 ± 78.0 to 503.9 ± 58.1 m in the anti-M2-R (−) group and from 195.9 ± 83.2 to 463.1 ± 56.1 m in the anti-M2-R (+) group. The structure and function of the left ventricle and 6-min walk distance improved greatly in the first year, especially in the first half of the first year. It is worth noting that anti-M2-R (−) patients showed greater improvement than anti-M2-R (+) patients. LVEF returned to normal in 89.3% (92/103) of the patients during the first year and 94.2% (97/103) of patients during the second year.

### Primary endpoint events

**Fig. 4:**
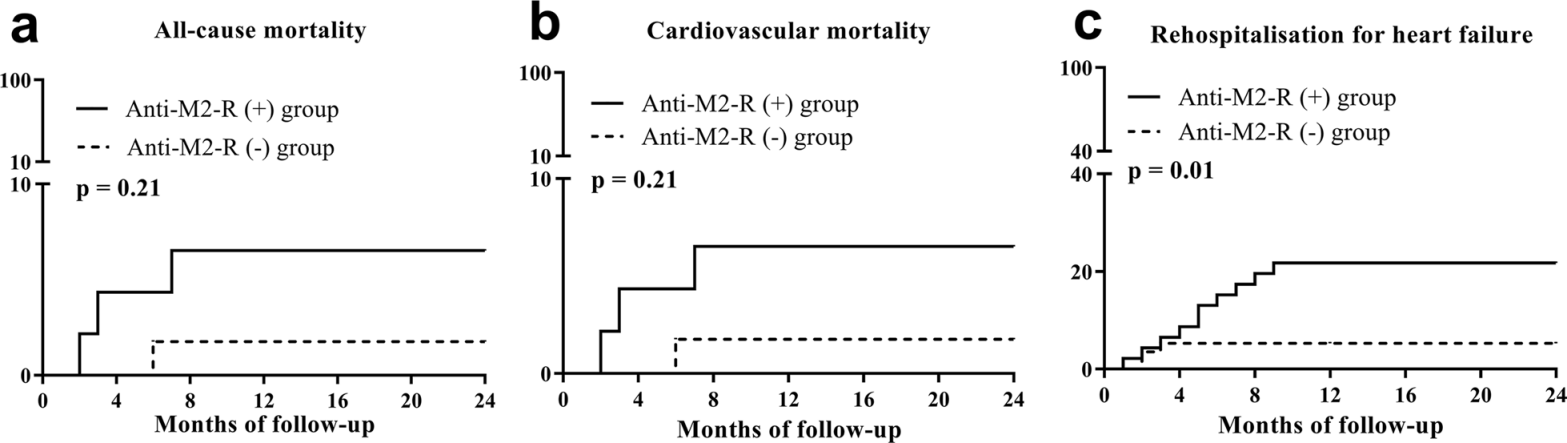
Endpoint events in both groups. **a** All-cause mortality, **b** cardiovascular mortality, and **c** rehospitalisation for heart failure

During the follow-up, four patients died during hospitalisation due to the progression of HF. One patient was in the anti-M2-R (−) group, and the other three patients were in the anti-M2-R (+) group. There were no differences in all-cause mortality or cardiovascular mortality between the two groups (*p* = 0.21). Thirteen patients were rehospitalised for acute exacerbation of HF, including three patients in the anti-M2-R (−) group and 10 patients in the anti-M2-R (+) group (*p* = 0.01), as shown in Fig. 4. Multivariate analysis was conducted to evaluate the effect of the following baseline variables on rehospitalisation for HF: anti-M2-R (positive vs. negative), dosages of metoprolol, LVEDD, LVEF (≤ 35% vs. > 35%), NT-proBNP (> 4500 pg/mL vs. ≤ 4500 pg/mL). Among these, positive for anti-M2-R was the independent factor for rehospitalisation for HF (Table 3).

**Table 3:** Univariate and multivariate analysis for rehospitalisation for HF

	Univariate analysis			Multivariate analysis		
	OR	95% CI	*p* value	OR	95% CI	*p* value
LVEDD	1.1	1.02–1.20	0.015			
LVEF	9.7	2.7–35.1	0.001	5.1	1.2–21.4	0.026
NT-proBNP	8.6	1.8–41.3	0.007	7.6	1.4–41.4	0.019
Dosages of metoprolol	0.88	0.79–0.98	0.015			
Anti-M2-R	5.0	1.3–19.4	0.020	5.2	1.1–23.6	0.033

*OR* odds ratio, *CI* confidence interval, *LVEDD* left ventricular end-diastolic diameter, *LVEF* left ventricular ejection fraction, *NT-proBNP* N-terminal pro-brain natriuretic peptide, *Anti-M2-R* autoantibodies against the M2-muscarinic receptor

## Discussion

### Major findings

In this study, the clinical outcomes of 103 PPCM patients who received standard treatment regimens for HF were analysed. The five major findings were as follows: (1) the heart rate of anti-M2-R (+) patients was lower than that of anti-M2-R (–) patients. (2) Patients in the anti-M2-R (−) group required higher doses of metoprolol and digoxin compared with patients in the anti-M2-R (+) group. (3) With the same dosage of digoxin, the mean SDC was higher in anti-M2-R (+) patients than in anti-M2-R (−) patients. (4) LVEF ≥ 50% was observed in 89.3% (92/103) of patients at the 1-year mark and in 94.2% (97/103) of patients at the 2-year mark. Anti-M2-R (−) patients showed a greater reduction of the left ventricular diameter and more improvement in cardiac function compared with anti-M2-R (+) patients. Multivariate analysis identified negativity for anti-M2-R as the independent factor for cardiac function improvement. (5) Four patients died due to the progression of HF. Risk of rehospitalisation for HF decreased in the anti-M2-R (−) group. However, there were no differences in all-cause mortality or cardiovascular mortality between the two groups.

### Anti-M2-R and PPCM

The M2 receptor is the main muscarinic acetylcholine receptor expressed on cardiomyocytes [8]. Anti-M2-R was first detected in patients with idiopathic dilated cardiomyopathy (IDCM) [6]. Anti-M2-R can also be detected in patients with multiple causes of HF, such as ischemic cardiomyopathy, hypertensive heart disease, rheumatic valvular heart disease, and PPCM. The positive rate and titres of this antibody in PPCM patients were similar to those in patients with other aetiologies of HF [3, 9]. An animal study shows that serum anti-M2-R is related to cardiac structural and functional changes. It can induce ventricular enlargement and the thinning of the walls—the typical changes of IDCM and PPCM in humans—by monthly immunisation peptides in accordance with the sequence of the second extracellular loop of the M2 receptor in rabbits [10, 11]. Immunisation by using plasmid DNA encoding entire M2 receptor proteins or epitopes could lead to cardiac remodelling, contractile dysfunction, and autophagy in the myocardium in mice [12, 13]. Previously, we conducted a clinical study on patients with familial dilated cardiomyopathy with C722G mutation who were positive for anti-M2-R, and we found these patients had more progressive disease, characterised by sudden death, arrhythmia, and HF [14]. We also found anti-M2-R negative HF patients had greater improvements than anti-M2-R positive patients, and this was accompanied by a marked decrease of rehospitalisation for HF [15]. In this study, we mainly explored the role of anti-M2-R in the treatment of PPCM patients and found similar results. Further studies are needed for clarification.

### Regulation of the autonomic system

The autonomic system has an important influence on the progression of PPCM. Elevated activities of the sympathetic system are associated with an adverse prognosis. Activation of the vagus nerve system seems to be a double-edged sword. With the extensive use of ACEIs and β-receptor blockers, the activities of the sympathetic system were mainly attenuated, but the effects of the standard pharmacological regimen in several patients were poor. For these patients, the tension of the vagus nerve system may have been activated pathologically. In anti-M2-R (+) patients, the chronic interaction between anti-M2-R and the M2 receptor caused a pathological activation of the cardiac vagus nerve system. Therefore, these patients showed a slower heart rate and lower maximum tolerated dose of metoprolol compared with anti-M2-R (−) patients. In the anti-M2-R (−) group, the enlarged left ventricular chamber could return to normal more rapidly. Positive for anti-M2-R was the independent factor for  rehospitalisation for HF in the multivariate analysis.

### The prognosis of PPCM

The standard treatment of PPCM is based on anti-HF treatment, which focuses on controlling symptoms, inhibiting excessive activation of the neuroendocrine system, and preventing thromboembolism and cardiac arrhythmia. The prognosis of PPCM is better than that of other cardiomyopathies with reduced LVEF, which may be linked to the removal of hormonal toxins by the delivery of placenta and the termination of lactation. Reports on the prognosis of PPCM differ over time and geographic location. In Haiti, Turkey, and South Africa, only 21–43% of PPCM patients return to normal LVEF, and the mortality rates in these countries are 15–30%, 30%, and 28–40%, respectively [16]. A study of 85 PPCM patients that included follow-ups for up to 7 years showed that 16% (14/85) of the patients died of PPCM or related problems in the United States [17]. An IPAC study enrolled 100 PPCM patients from 30 centres in North America. After 1 year of treatment, the LVEF of 72% of the PPCM patients returned to normal, and 12 patients had major cardiovascular events, including four deaths [18]. A worldwide registry study retrospectively enrolled 411 PPCM patients, and there were 10 deaths in 1 month, including six deaths from HF, three from sudden cardiac events, and one from stroke [19]. In this study, the LVEF of 89.3% and 94.2% of PPCM patients returned to normal at the 1-year and 2-year marks, respectively, and the 2-year mortality rate was 3.9%, which was similar to or better than the 2-year mortality rates in other reports.

### Limitations

There were some limitations in this study. Firstly, there was a relatively small number of patients with the specified conditions in the two hospitals in the past 20 years; moreover, these patients were almost all from Northern China and Eastern China, so there was a lack of regional differences and ethnic diversity amongst the subjects. Secondly, due to factors such as population movement, urban demolition, and changes in contact information, four patients were lost to follow-up during the first year. Thirdly, with the continuous updating of HF management in the past 20 years, the treatment strategies for PPCM patients also evolved. Fortunately, the vast majority of cases in this study were selected between 2000 and 2018, and most of them received standard neuroendocrine blockade therapy.

## Conclusions

There were no differences in all-cause mortality or cardiovascular mortality between the two groups. The rehospitalisation rate for HF decreased in the anti-M2-R (−) group. This difference may be related to the regulation of the autonomic nervous system by anti-M2-R.

## Data Availability

All of the data are available upon reasonable request from the corresponding author.

## Abbreviations

ACEI: Angiotensin-converting enzyme inhibitors
Anti-β1-R: Autoantibodies against β1-adrenergic receptors
Anti-M2-R: Autoantibodies against the M2-muscarinic receptor
CI: Confidence interval
HF: Heart failure
HFmrEF: Heart failure with midrange ejection fraction
HFrEF: Heart failure with reduced ejection fraction
IDCM: Idiopathic dilated cardiomyopathy
LVEDD: Left ventricular end-diastolic diameter
LVEF: Left ventricular ejection fraction
LVESD: Left ventricular end-systolic diameter
NT-proBNP: N-terminal pro-brain natriuretic peptide
NYHA: New York Heart Association
OR: Odds ratio
PPCM: Peripartum cardiomyopathy
RAAS: Renin–angiotensin–aldosterone system
SDC: Serum digoxin concentration
